# Cross-Reactive and Lineage-Specific Single Domain Antibodies against Influenza B Hemagglutinin

**DOI:** 10.3390/antib8010014

**Published:** 2019-02-10

**Authors:** Walter Ramage, Tiziano Gaiotto, Christina Ball, Paul Risley, George W. Carnell, Nigel Temperton, Chung Y. Cheung, Othmar G. Engelhardt, Simon E. Hufton

**Affiliations:** 1 Biotherapeutics Division, National Institute for Biological Standards and Control, Blanche Lane, South Mimms, Potters Bar, Hertfordshire EN6 3QG, UK; walter.ramage@nibsc.org (W.R.); Tiziano.Gaiotto@gmail.com (T.G.); chris.ball@nibsc.org (C.B.); paul.risley@nibsc.org (P.R.); 2 Infectious Diseases and Allergy Group, School of Pharmacy, University of Kent, Kent ME4 4TB, UK; g.carnell@gmail.com (G.W.C.); n.temperton@kent.ac.uk (N.T.); 3 Division of Virology, National Institute for Biological Standards and Control, Blanche Lane, South Mimms, Potters Bar, Hertfordshire EN6 3QG, UK; chung.cheung@hli.ubc.ca (C.Y.C.); othmar.engelhardt@nibsc.org (O.G.E.)

**Keywords:** influenza, influenza B virus, hemagglutinin, single domain antibody, Nanobody^TM^, phage display, yeast display, epitope mapping

## Abstract

Influenza B virus (IBV) circulates in the human population and causes considerable disease burden worldwide, each year. Current IBV vaccines can struggle to mount an effective cross-reactive immune response, as strains become mismatched, due to constant antigenic changes. Additional strategies which use monoclonal antibodies, with broad reactivity, are of considerable interest, both, as diagnostics and as immunotherapeutics. Alternatives to conventional monoclonal antibodies, such as single domain antibodies (Nanobodies^TM^) with well-documented advantages for applications in infectious disease, have been emerging. In this study we have isolated single domain antibodies (sdAbs), specific to IBV, using alpacas immunised with recombinant hemagglutinin (HA) from two representative viruses, B/Florida/04/2006 (B/Yamagata lineage) and B/Brisbane/60/2008 (B/Victoria lineage). Using phage display, we have isolated a panel of single domain antibodies (sdAbs), with both cross-reactive and lineage-specific binding. Several sdAbs recognise whole virus antigens, corresponding to influenza B strains included in vaccines spanning over 20 years, and were capable of neutralising IBV pseudotypes corresponding to prototype strains from both lineages. Lineage-specific sdAbs recognised the head domain, whereas, sdAbs identified as cross-reactive could be classified as either head binding or stem binding. Using yeast display, we were able to correlate lineage specificity with naturally occurring sequence divergence, at residue 122 in the highly variable 120 loop of the HA1 domain. The single domain antibodies described, might have applications in IBV diagnostics, vaccine potency testing and as immunotherapeutics.

## 1. Introduction

Seasonal influenza caused by the influenza A virus (IAV) and the influenza B virus (IBV) remains a considerable public health challenge [1]. IBV has received less attention than IAV, largely because it does not cause pandemics as there are no naturally occurring, non-human reservoirs to facilitate the extensive antigenic changes, characteristic of a pandemic. However, there has been a recent increase in the rate of IBV infection, globally, with between 20–30% of the total influenza burden now due to influenza B [2]. Current vaccines can struggle to induce sufficient levels of cross-protective immunity against IBV and strains often become mismatched, due to the constant antigenic changes in the influenza virus [3]. The virus also co-circulates as two antigenically distinct lineages and, therefore, the dominant circulating lineage must be predicted for inclusion in the trivalent vaccine. This highlights the need to develop additional approaches to both treat and protect from the influenza B virus infection. Passive immunotherapy, using broadly neutralising antibodies against the major viral coat protein hemagglutinin (HA), is one approach that has shown great promise for the treatment of IAV infection [4]. The precursor HA protein (HA0) is cleaved by the host proteases into a form that comprises a highly variable distal head domain, HA1, and a more conserved proximal stem region, HA2 [5]. Most broadly, neutralising anti-HA monoclonal antibodies that have been described to date, target the HA stem of IAV [6,7,8]; however, monoclonal antibodies with broad reactivity have been described, which bind the more variable head domain, using a single CDR3 loop for antigenic recognition [9]. The more recently reported broadly neutralising monoclonal antibodies against IBV have also shown the presence of conserved epitopes, in both the stem region and the head domain [10,11,12]. A feature common between the broadly neutralising human monoclonal antibodies against both IBV and IAV, is the low levels of somatic hypermutation and ‘heavy-chain-only’ binding [7,10]. ‘Heavy-chain-only’ binding has been suggested to be the preferred mode of binding to influenza HA, as has been described for HIV [13]. These observations highlight that the VL domain might not be required for binding occluded epitopes onto the HA stem. Guided by these observations, we have isolated naturally occurring ‘heavy-chain-only’ antibodies from camelids (also known as Nanobodies^TM^), believing their small size, single domain structure and long CDR3 loops make this unique antibody format well-equipped to access similar HA stem epitopes [14,15,16].

In the 1980’s, the influenza B virus diverged to give two distinct lineages, termed the B/Yamagata/16/88 and B/Victoria/2/87 lineages [17]. Although the HA stem region remains highly conserved, the head domain has diverged to become sufficiently antigenically and genetically distinct, to warrant an independent inclusion in the current quadrivalent influenza vaccines [18]. The inclusion of seasonal strains from both IBV lineages, in turn, presents a challenge for both vaccine production and potency determination. Separate monoclonal antibodies, with either B-Victoria or B-Yamagata lineage-specific recognition could find applications in determining the individual antigenic content of each IBV lineage strain included in the seasonal vaccines [19]. However, the challenge in identifying lineage-specific binding antibodies is that, such epitopes are expected to be in the more variable head domain of the HA and, as such, are susceptible to antigenic change with associated loss of reagent binding. The degree of antigenic divergence, tolerated by a given monoclonal antibody binding to the variable head domain, which is subject to constant selective pressure to change, to some extent, is determined by the size of the epitope footprint. It is interesting to speculate that a small epitope footprint could be an advantage, as there may be less amino acid mutations that can impact antibody reagent binding. Single domain antibodies isolated from camelids, have preferences for smaller crevices and clefts on protein surfaces, as compared to the conventional two-chain monoclonal antibodies, which generally bind to larger flatter epitopes [20,21]. As such, single domain antibodies (sdAbs) may have a smaller epitope footprint and, therefore, might be able to retain binding to a constantly changing virus, for longer. In addition, human monoclonal antibodies to the HA head domain derived from either naturally infected or vaccinated patients might have already contributed to the antigenic pathways of viral escape and might be pre-ordained to the loss of binding. For these reasons, we have chosen to isolate the IBV lineage-specific sdAbs, rather than the conventional MAbs (Monoclonal Antibodies), believing that this unique antibody format could also form the basis of a robust and durable immunoassays for potency determination of the IBV containing vaccines [19].

By immunising alpacas with recombinant HA and the selection of phage display libraries, we have isolated sdAbs with both cross-reactivity and lineage-specific recognition. Through combining conventional phage display selections and next-generation sequencing, we have isolated rare sdAbs with either B-Victoria or B-Yamagata lineage specificity. We have grouped sdAbs, on the basis of head or stem binding, and used yeast display, to further de-lineate an epitope associated with B-Victoria lineage-specific recognition. The potential applications of these different classes of sdAbs for applications in the IBV vaccine potency assays and as immunotherapeutics, are also discussed.

## 2. Materials and Methods 

### 2.1. Influenza Antigens and Immunisation of the Alpacas

The virus antigen standards used in this study were B/Brisbane/60/2008 (National Institute for Biological Standards and Control (NIBSC) 14/146), B/Malaysia/2506/2004 (NIBSC 08/184), B/Shangdong/9/97 (NIBSC 04/128), B/Victoria/2/87 (NIBSC 87/774) of the B-Victoria lineage; the B/Yamagata/16/88 (NIBSC 92/628), B/Yamanashi/166/98 (NIBSC 99/586), B/Harbin/7/94 (NIBSC 97/748), B/Jiangsu/10/2003 (NIBSC 04/202), B/Florida/4/2006 (NIBSC 08/140), B/Massachusetts/02/2012 (NIBSC 13/106), B/Phuket/3037/2013 (NIBSC 14/252), B/Utah/9/2014 (NIBSC 15/100) of the B-Yamagata lineage; and the pre-lineage split strain B/HongKong/8/73 (NIBSC 79/568). Seasonal H1N1 strain A/Brisbane/10/2007 (NIBSC 08/278) was used as a negative control. Purified recombinant hemagglutinins used in this study were B/Brisbane/60/2008 (B-Victoria) and B/Florida/04/2006 (B-Yamagata) (Protein Sciences^TM^, Meriden, CT, USA). Purified HA1 head domains from B/Brisbane/60/2008 (B-Victoria) and B/Florida/04/2006 (B-Yamagata) were also used (Sino Biologicals^TM^, Beijing, China). Juvenile male alpacas were purchased through the Royal Veterinary College, Hertfordshire, UK. All experiments were reviewed by a local ethics committee and performed under a U.K Home Office licence PPL 80/2581. A blood sample, prior to immunization, was obtained from the external jugular vein and this was followed by four intramuscular injections on day 0 (primary immunisation), 21, 43 and 71, with injections being administered to the rear legs (thigh region) on days 0 and 43, and to the front legs (thigh region) on days 21 and 71. The primary immunisation consisted of 50 µg of recombinant HA B/Brisbane/60/2008 (B-Victoria) or B/Florida/04/2006 (B-Yamagata) (ProteinSciences^TM^) in 400 μL of sterile PBS (phosphate buffered saline), which was emulsified with 800 μL of Freund’s complete adjuvant (Sigma Aldrich, St. Louis, MO, USA), just prior to immunisation. Similarly, the three booster injections of 50 μg of the same recombinant HA as the primary immunisation in Freund’s incomplete adjuvant (Sigma), were administered. Approximately four days after each injection, a 10 mL blood sample was collected from the external jugular vein, from which the serum was prepared, after allowing the blood to clot overnight at 4 °C.

### 2.2. Construction and Selection of the Phage-Displayed Libraries

For the antibody library construction, approximately 10 mL blood samples were collected into the heparinised tubes, from an immunised alpaca. Peripheral blood mononuclear cells were purified using a Ficol Hypaque centrifugation procedure (Sigma) and RNA was extracted using a RiboPure^TM^ RNA extraction kit (Novagen, Thermo Fisher Scientific, Waltham, MA, USA) following the manufacturer’s instructions. First strand cDNA synthesis was performed using Superscript III^TM^ reverse transcriptase (Invitrogen, Thermo Fisher Scientific, Waltham, MA, USA) and oligo-dT primer with 200 ng of total RNA per reaction. Primary PCR and secondary PCR to recover the alpaca *VHH* genes (DNA encoding the variable region of the heavy-chain-only antibodies) appended with *Sfi*1 and *Not*1 restriction sites were carried out, as previously described [14]. Approximately 5 µg of the VHH antibody DNA was digested by the *Not*1 and *Sfi*I restriction enzymes (New England BioLabs, Ipswich, MA, USA) and then ligated into a phage-display vector, pNIBS-1, which contains a His tag for sdAb purification, a Myc tag for detection and a suppressible stop codon for soluble expression [14]. After purification (Qiagen, Hilden, Germany), the ligation mix was transformed into the TG1 electro-competent cells (Agilent, Santa Clara, CA, USA), using electroporation. Phage antibody library selections were performed using immunotubes (Nunc, Thermo Fisher Scientific, Waltham, MA, USA) coated overnight, at 4 °C, with 1 mL of the 10 µg/mL recombinant HA (Protein Sciences^TM^) in PBS or the whole virus antigen standards reconstituted in PBS [14].

### 2.3. Antibody Expression and Screening

Individual colonies from each round of selection were picked and grown overnight, at 37 °C, in 2 × TY supplemented with carbenicillin (100 µg/mL) and 2.0% (*w*/*v*) glucose and plasmid DNA was isolated (Qiagen, Hilden, Germany). Cloned VHH genes were sequenced, aligned and grouped, according to the CDR3 length and homology. CDR3 sequences of high homology and identical length were assumed to have been derived from clonally related B cells and were likely to bind to the same, or overlapping, epitope on HA. Representative members from each proposed epitope group were expressed and purified. For single domain antibody production, pNIBS-1 clones were transformed into the non-suppressor *Escherichia coli* strain WK6 (New England Biolabs). Soluble antibody expression was induced with the addition of IPTG to the 1 mM final concentration, followed by a further incubation, overnight, at 30 °C. Periplasmic extracts were prepared [14] and purified by immobilised metal chelate chromatography (IMAC), using Ni-NTA spin columns (Qiagen) or TALON^TM^ resin (Clontech, Takara Bio Inc., Mountain View, CA, USA), according to manufacturer’s instructions, depending on the scale. Purified samples were then dialysed, using Slide-A-Lyzer cassettes with a 3.5 kDa molecular weight cut-off (Pierce, Thermo Fisher Scientific, Waltham, MA, USA) into the PBS and the size and purity assessed by the SDS-PAGE. Purified single domain antibodies were screened for binding to the recombinant HAs and to the influenza virus antigen standards. Influenza virus antigen standards (National Institute for Biological Standards and Control, NIBSC) were reconstituted in 1 mL sterile water and then diluted 1/20 in PBS (prior to incubation) overnight, at 4 °C, in a 96-well plate (Nunc), followed by ELISA using an HRP (horseradish peroxidase) conjugated anti-c-Myc secondary reagent and TMB (3,3’,5,5’ tetramethylbenzidine) detection at OD_450_nm [14].

### 2.4. Analysis Using Surface Plasmon Resonance

For binding and affinity ranking, a BIAcore T100 machine (GE Healthcare, Marlborough, MA, USA) was used, in combination with a single-cycle kinetics procedure [22]. In brief, the purified recombinant hemagglutinins from different Influenza B viruses were immobilised onto a BIAcore^TM^ CM5 chip in 10 mM sodium acetate pH 5.5, using an amine coupling kit (GE Healthcare), to approximately 3000 RU. A concentration series from 1–100 nM of purified sdAbs were run over the different antigen surfaces. A reference surface was subtracted, prior to evaluation of the sensorgrams, using the single-cycle kinetics procedure of the BIAevaluation^TM^ software (GE Healthcare) and a 1:1 fitting model. Binding the full length HA0 or the head domain, HA1, of hemagglutinin was evaluated using the recombinant B-Victoria HA0, B/Brisbane/60/2008, and B-Yamagata HA0, B/Florida/04/2006, (Protein Sciences^TM^) or B-Victoria HA1, B/Brisbane/60/2008, and B-Yamagata HA1, B/Florida/04/2006 (Sino Biological Inc., Beijing, China).

### 2.5. Next-Generation-Sequence-Assisted Single Domain Antibody Discovery

**Figure 1:**
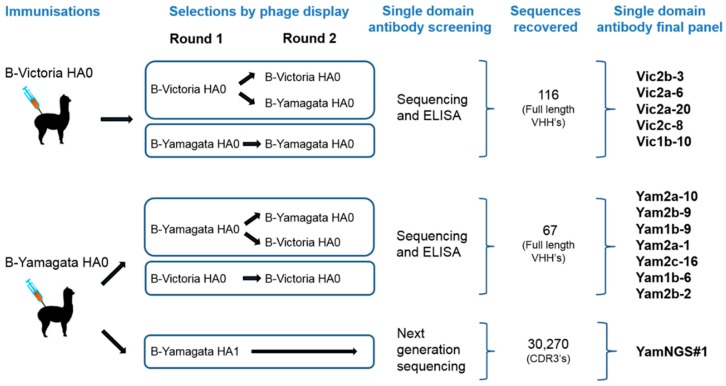
Selection strategies to isolate cross-reactive and lineage-specific single domain antibodies (sdAbs) against influenza B hemagglutinin. The figure shows the immunisation approaches, selection and screening strategies. The number of VHH CDR3 sequences isolated from each approach is indicated.

Plasmid DNA was extracted from *E. coli* cultures grown from pre- and post-selection libraries (Figure 1), to obtain template DNA for the next-generation sequencing (NGS). A primary PCR reaction was performed using Phusion Hot Start II High Fidelity Polymerase (Thermo Fisher Scientific) and the primers: NGS_Alp_Fr1_Q (5′-tcgtcggcagcgtcagatgtgtataagagacagCAGCCGGCCATGGCACAG-3′) and NGS_FR4_Rev_AD (5′-gtctcgtgggctcggagatgtgtataagagacagTGAGGAGACGGTGACCTG-3′), which encoded the VHH gene flanking plasmid sequence (upper case) and adaptor sequences for the Nextera XT indexing (lower case), resulting in PCR products between 450 bp and 550 bp. The PCR products were purified (Qiagen) and used as a template for a secondary low-cycle number indexing PCR, using a Nextera XT indexing kit (Illumina, San Diego, CA, USA). Resulting PCR products were purified (Qiagen), quantified using the Qubit 2.0 fluorimeter (Thermo Fisher Scientific) and the DNA1000 Kit (Agilent Technologies, Santa Clara, CA, USA), followed by quality checking, using the Bioanalyzer 2100 (Agilent Technologies). Samples were prepared and multiplexed for use with the MiSeq reagent kit v3 (2 × 300 cycle) and run on the Illumina MiSeq, according to the manufacturer’s protocol. The de-multiplexing and trimming of sequences were performed on the Illumina MiSeq instrument. Copy numbers of the individual CDR3′s were obtained from the reverse read FastQ files, using the “antibody mining toolbox” [23]. Our intention at this stage was not a full-length sdAb repertoire analysis but to identify the unique high frequency CDR3′s, which were highly enriched by the selection of Influenza B HA1 domain, compared to their frequency in the original phage library. Relative frequency of each CDR3, as a percentage of the total, was determined by dividing the individual CDR3 copy number by a total CDR3 copy number ×100 (%RF) and the enrichment level (fold) was determined by the CDR3 %RF, after selection, divided by the CDR3 %RF, in the absence of any selection (the original phage library). Candidate CDR3′s from the selections on recombinant HA1 that showed high levels of enrichment, were compared with the enrichment levels of the same CDR3′s in the selections of full-length HA0. Full-length DNA sequence for four sdAbs containing these highly enriched candidate CDR3′s were then obtained, using the DNA sequence of the CDR3 of interest, to search the reverse read FastQ file using the Geneious 7.1.2 (https//www.geneious.com), combining the sequences found with the corresponding forward reads from the forward FastQ file. Sequences were synthesised (IDT), the sdAbs were expressed and purified, as before, and the binding specificities were determined by ELISA.

### 2.6. Lentiviral Pseudotype Assays

Lentiviral pseudotypes were produced by transient co-transfection of HEK293T/17 cells, using polyethylenimine [24,25]. Plasmid p8.91 encodes the structural (gag, pol) genes, whereas, pCSFLW represents the genome incorporated into the pseudotypes, bearing the firefly luciferase reporter. Influenza B hemagglutinin genes, in the expression plasmid pI.18, were also added to this mix, alongside the Human Airway Trypsin (HAT) expression plasmid, pCAGGS-HAT to allow for HA0 to HA1/2 maturation. The pseudotype-based microneutralisation assay (pMN) was carried out in Nunc^TM^ F96 microplates (Thermo Fisher Scientific) [24,25]; 1:2 serial dilutions were performed with the relevant sdAbs, across the 96-well plate in a total of 50 µL DMEM (10% foetal bovine serum, 1% penicillin/streptomycin). HIV-1-derived lentiviral pseudotypes, bearing the influenza B HA of choice, was then added to yield a relative luminescence unit (RLU) input of 1.5 × 10^6^ per well, in a total volume of 50 µL [26]. The plates were then incubated in a humidified incubator at 37 °C, 5% CO_2_, for one hour, after which 1 × 10^4^ HEK293T/17 cells were added, per well, in a total volume of 50 µL. After 48 h, the supernatants were removed and a 50:50 mix of PBS and Bright-Glo^TM^ (Promega, Madison, WI, USA) was added to each well. Plates were incubated at room temperature, for five minutes, and then luminescence was read using a Glomax^®^ luminometer (Promega). Results were normalised to cell and virus only controls, representing 100% and 0% neutralization, respectively, and IC_50_ values were calculated by non-linear regression, using GraphPad 5 Prism.

### 2.7. Construction and Screening of a Randomly Mutagenised HA Library Using Yeast Display

The coding sequence of the Influenza B virus hemagglutinin (HA0) of the strain B/Brisbane/60/2008 (GenBank FJ766840.1, minus the N-terminal signal sequence and the C-terminal transmembrane domain, comprising amino acids D1-I534) was codon-optimised for expression in yeast and synthesised, with 5′ *Sfi*I and 3′ *Not*I restriction sites (IDT). The construct was cloned as a *Sfi*I/*Not*I restriction fragment into the yeast display vector pNIBS-5 [16], which carried an SV5 tag, and transformed into *Saccharomyces cerevisiae* EBY100 (Invitrogen), using a yeast transformation kit (Sigma). 

A library of the HA0 mutants was generated by error-prone PCR, at a low error rate, to give approximately, 1 mutation per HA gene [16]. Approximately 20 µg of the HA0 error-prone PCR product was co-transformed with 20 µg of the *Sfi*1/*Not*1 digested pNIBS-5 vector, into the EBY100 competent cells, for a recombination between the PCR products and the yeast vector to take place in the yeast cells [16]. Standard procedures and recipes for growth, induction, yeast cell labelling, media and buffer preparation were used [16]. Staining for the sdAb binding to HA0 was performed by incubating yeast cells with 200 nM of purified sdAbs (c-Myc tagged), followed by a chicken anti c-Myc antibody (Bethyl Laboratories, Montgomery, TX, USA), and then by a goat-anti-chicken IgG AlexaFluor647-labelled secondary antibody (Jackson ImmunoResearch Europe, Ely, UK). Staining of yeast cells for HA0 display was performed by incubating yeast cells with mouse monoclonal anti-SV5 antibody (AbSerotec, Bio-Rad, Hercules, CA, USA), followed by a goat anti-mouse IgG AlexaFluor488-labelled secondary antibody (Thermo Fisher scientific). Staining was performed simultaneously for the sdAb binding and the HA0 display. The mutant yeast library was grown in a selective medium for induction of the HA display and, approximately, 10^6^ cells were co-stained with purified sdAbs at 200 nM, followed by anti-SV5 and anti-c-Myc detection antibodies, and then by fluorescent secondary antibodies, as above [16]. Flow cytometric cell sorting was performed using the BDAria III and FACSDiva software v8.0.1 (Becton Dickenson, Franklin Lakes, NJ, USA). A sorting gate was chosen to sort cells for the display of HA0 (by virtue of the anti-SV5 signal), but also for the absence of sdAb binding. A second round was then performed using the same conditions. Yeast DNA minipreps were made from the final round of sorting and transformed into the TG1 electro-competent *Escherichia coli* cells (Agilent). Then, plasmids from single bacterial colonies were sequenced. Resulting sequences were aligned to wt HA0 (wild-type B/Brisbane/60/2008 HA0), using Geneious 7.1.2 (https//www.geneious.com) to identify candidate mutations. Plasmids containing single residue candidate mutations were re-created, where required, using the QuikChangeII site directed mutagenesis kit (Agilent) and re-transformed into yeast cells. Individual yeast clones containing either a mutant HA0 or a yeast containing the wt HA0, were grown in a selective medium, for induction of the HA display and cells stained for display and sdAb binding, as above. Binding of each sdAb to each mutant HA0 and wt HA0 yeast clone was determined by flow cytometry on a FACS CantoII flow cytometer (Becton Dickinson). Data collection and analysis was performed using the FACSDiva and FlowJo v10, (LLC, Ashland, OR, USA) software.

## 3. Results

### 3.1. Isolation and Characterisation of Cross-Reactive and Lineage-Specific Single Domain Antibodies against the Influenza B Hemagglutinin

Two phage displayed sdAb libraries were constructed from purified peripheral blood mononuclear cells of alpacas immunised with recombinant HA0 from the representative B-Victoria (B/Brisbane/60/2008) or B-Yamagata (B/Florida/04/2006) lineage viruses. The size of the B-Victoria library was 9.4 × 10^7^ independent clones and the B-Yamagata library was 3.7 × 10^7^ independent clones. Each phage library was selected for two rounds on the HA antigen used for immunization, for an unbiased recovery of all sdAbs binding to the immunogen. To bias for the selection of cross-reactive sdAbs, a second strategy was also performed, by alternating selections between the B-Victoria lineage and the B-Yamagata lineage antigens (Figure 1). 

After selection, sdAbs were sequenced and grouped into clonally related families, based on the CDR3 sequence length and similarity. Single domain antibodies with identical CDR3 length and high sequence similarity were assumed to be clonally related and, thus, binding to the same or overlapping epitopes, so a single representative clone from each family was taken forward for further analysis.

**Figure 2:**
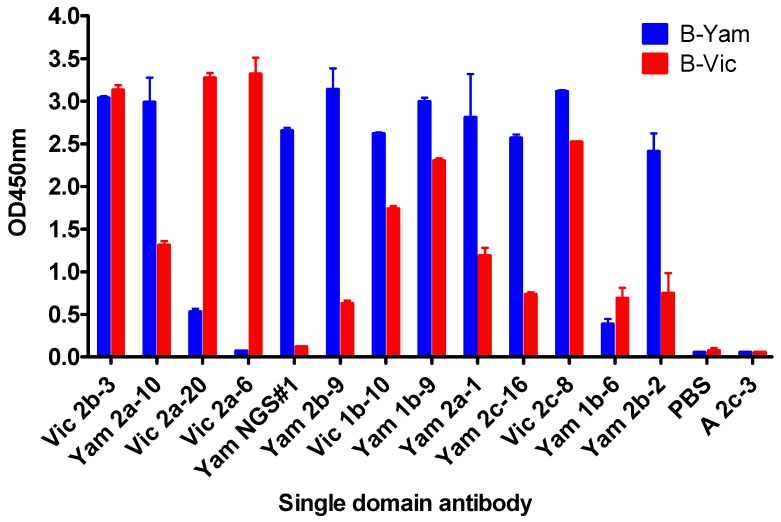
ELISA showing sdAb binding to the recombinant hemagglutinin (HA0). sdAb binding to the recombinant HA0 from Influenza B-virus lineage representative strains used in immunisations and phage display library selections. B-Vic is B/Brisbane/60/2008, B-Yam is B/Florida/04/2006. Control sdAb A2c-3 is specific for influenza A hemagglutinin, PBS is a no sdAb negative control. ELISA signal is an average of 2 assays.

Our ELISA screening identified sdAbs with a pre-dominantly cross-lineage reactivity (Figure 2), which was not surprising, given the high sequence homology between the B-Yamagata and the B-Victoria strains.

In addition to cross-lineage reactive sdAbs, we were able to identify Vic2a-6 with B-Victoria lineage-specific recognition, however, we were unable to isolate any B-Yamagata lineage-specific sdAbs, using the conventional screening. We reasoned that epitopes defining B-Yamagata lineage specificity would be rare and likely to bind to the more variable HA1 head domain [5]. Therefore, we selected the B-Yamagata phage display library on a purified recombinant HA1 domain of B/Florida/4/2006, to bias against the stem reactive sdAbs, which had dominated previous selection strategies, and then used next-generation sequencing to identify new unique CDR3 sequences [27]. Highly enriched CDR3 sequences were identified, relative to the unselected CDR3 pool, and compared to those sequences already recovered, using conventional phage display selections. This led to the identification of several VHH CDR3 sequences which had not previously been identified (Figure S1). Full-length VHH sequences were synthesised and four candidate sdAbs were expressed and tested for B-Yamagata lineage-specific recognition, resulting in one unique B-Yamagata lineage-specific sdAb, YamNGS#1. A final panel of thirteen unique sdAbs were taken forward, which included eleven cross-lineage reactive sdAbs, one B-Victoria lineage, and one B-Yamagata lineage-specific sdAb (Table 1) (Figure 1). All sdAbs were shown to have the characteristic camelid heavy-chain-only antibody framework 2 “hallmark” residue substitutions [28]. CDR3 lengths varied from 5 to 20 residues (Table 1) and all sdAbs were expressed at yields between 3 mg/L to 27 mg/L.

We tested the extent of cross-reactivity on a panel of whole virus antigen standards, corresponding to the strains included in seasonal vaccines between 1973 and 2014 (Table 2). Most of the sdAbs initially identified as cross-reactive with the B lineage representative strains B/Brisbane/60/2008 (B-Victoria) and B/Florida/04/2006 (B-Yamagata), used for library construction (Table 1), showed extensive cross-reactivity going, both, forward and backwards in time, including the pre-lineage split strain (B/HongKong/08/73).

Single domain antibodies Yam2b-9, Vic1b-10, Yam 1b-9, Yam2a-1, Yam2c-16, Vic2c-8, and Yam1b-6 recognised all antigen standards tested from the pre-lineage split virus B/HongKong/8/73, up to more recent vaccine viruses B/Brisbane/60/2008 (B-Victoria lineage) and B/Utah/9/2014 (B-Yamagata lineage). The B-Victoria lineage-specific sdAb Vic2a-6 showed reactivity to all B-Victoria strains tested between 1987–2008, but also bound the earliest B-Yamagata strain (B/Yamagata/16/1988), just post-split. YamNGS#1 was identified as having B-Yamagata lineage-specific binding and was able to recognize the Yamagata strains between 1994 and 2012, covering a duration of eighteen years (Table 2).

### 3.2. Grouping sdAbs on the Basis of HA1 Head Domain or Stem Domain-Specific Binding

Single domain antibodies were tested for binding to the head domain, or the HA stem region, using surface plasmon resonance on both full-length recombinant HA0 and HA1 (head domain) from representative B-Yamagata and B-Victoria strains (Table 3, Supplementary Figure S2).

Five sdAbs were shown to be specific to the HA1 domain and eight sdAbs only showed recognition of the full-length HA0. Most of the cross-reactive sdAbs were classified as stem-specific, however, three sdAbs were predicted to bind cross-reactive epitopes in the head domain. The B-Victoria lineage-specific sdAb Vic2a-6, bound to the head domain of the B/Brisbane/60/2008 (B Victoria), but did not bind to the B/Florida/04/2006 head domain or the full-length HA (B-Yamagata), which was consistent with recognition of a B-Victoria lineage-specific epitope in the head domain. YamNGS#1 bound to the head domain of the B/Florida/04/2006 (B-Yamagata), but showed no binding to the head domain of the B/Brisbane 60/2008 (B-Victoria), indicating that it binds to a B-Yamagata lineage-specific epitope in the head domain. The sdAbs had a high affinity down to <100 pM, reflecting the affinity maturation possible using the prime and boost immunisation of alpacas. The panel of sdAbs were grouped as cross-reactive head binding (Vic2b-3, Yam2a-10, Vic2a-20), cross-reactive stem binding (Yam 2b-9, Vic1b-10, Yam1b-9, Yam2a-1, Yam2c-16, Vic2c-8, Yam1b-6, Yam2b-2), B Victoria lineage-specific head binding (Vic2a-6) or B-Yamagata lineage-specific head binding (YamNGS#1) (Table 3).

### 3.3. In Vitro Neutralisation Activity of Single Domain Antibodies

Purified sdAbs were tested for their ability to neutralise influenza virus pseudotypes, corresponding to the different IBV lineages, using micro-neutralisation assays [24]. Six of the eight sdAbs identified as cross-reactive and binding to the epitopes in the HA stem region, were shown to be capable of neutralising pseudoviruses of both lineages with the most potent sdAb, Vic1b-10, having IC_50_ values of 0.2 nM and 15.5 nM on B-Vic and B-Yam lineage viruses, respectively (Table 4). Yam2b-9 and Yam2b-2, although cross-reactive on the recombinant antigen (Figure 2, Table 3), showed only a lineage-specific neutralisation activity. There was generally a good correlation between affinity and neutralisation activity, except for Vic2c-8, which was shown to be the least potent in neutralization, despite having an affinity comparable to the other cross-reactive, sdAbs (Table 3 and Table 4).

Both, Vic2a-6 and Vic2a-20 showed a B-Vic lineage-specific neutralisation activity with Vic2a-6 also showing a low level of neutralisation activity on the earliest post-split B/Yamagata/16/88 strain. This was consistent with Vic2a-6 recognising an antigen standard corresponding to the same strain (Table 2).

### 3.4. Identification of the B Victoria Lineage-Specific Epitope

**Figure 3:**
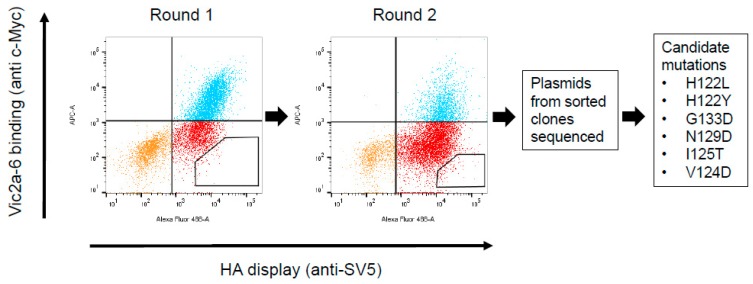
Cell sorting and isolation of candidate mutations interfering with the Vic2a-6 sdAb binding. Flow cytometry plots for two rounds of negative sorting for loss of binding to Vic2a-6, by gating cells in the lower right quadrant of the FACS dot plot, as indicated. Plasmids from the sorted yeast clones were sequenced and mutations in the HA which interfered with sdAb binding to HA, were identified. Residue numbering was according to B/HongKong/8/73 [29].

**Figure 4:**
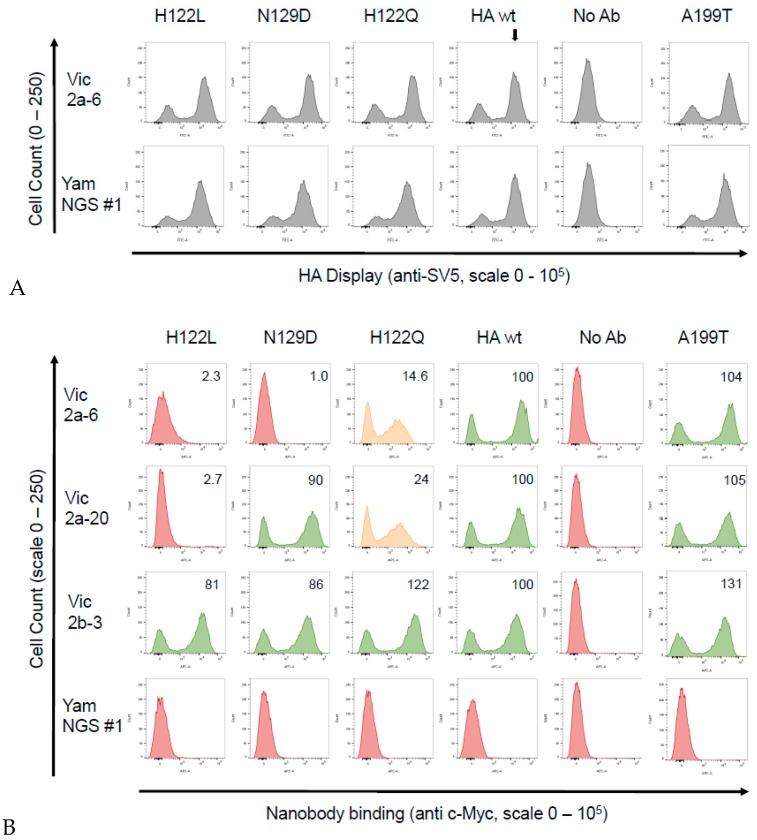
Binding of the sdAbs to the B-Victoria single residue mutant HA0’s. Candidate single residue mutations predicted to correlate with lineage-specific binding and a naturally occurring substitution H122Q, which differentiated between the B-Victoria and B-Yamagata strains, were generated and binding was tested using yeast display and flow cytometry. (**A**) Detection of the HA0 display level, using detection of the SV5 epitope tag attached to the HA0, showing that the mutations had no effect on the HA0 display level relative to the wild-type (wt) HA0 (indicated by vertical arrow). (**B**) Flow cytometry histograms showing binding of the Vic2a-6, Vic2a-20, Vic2b-3 and the YamNGS#1 to the wild-type HA (B/Brisbane/60/2008 precursor HA0) and selected mutants (the positive population is the right peak, whereas, the left peak shows the unbound and unstained populations). Mutations that completely eliminated sdAb binding are shown in red, those that partially affected binding are shown in orange and those that had no effect are shown in green. We determined the extent of sdAb binding by dividing the geometric mean fluorescence intensity (MFI) of each sdAb mutant pair by the value of the MFI of the wild-type B-Vic HA sdAb interaction and the resulting ratio, normalised to a percentage value of the wild-type interaction. Relative binding of the sdAbs was categorised as ‘no binding’ (in red)—< 5%; ‘intermediate binding’ (in orange)—between 20–40%; and ‘strong binding’ (in green)—> 40%; shown in the upper right-hand quadrant of the flow cytometry histograms. Graphs shown are a representative of at least two independent experiments.

For a more precise epitope mapping, we used the yeast surface display which we had previously used to epitope map the sdAb binding to HA from A(H1N1)pdm09 [16]. The B/Brisbane/60/2008 HA0 precursor gene (B-Victoria lineage) (corresponding to the residues D1-I534 of the mature protein) was sub-cloned into a yeast display vector, where it was fused to a SV5 epitope tag, enabling the detection of a full-length expression and display of the folded HA0 on the yeast surface (Figure 3). Yeast cells were simultaneously labelled with an anti-SV5 antibody to determine the HA0 display levels and an anti c-Myc antibody to show binding of the individual sdAbs. The head binding sdAbs Vic2b-3, Yam2a-10, Vic2a-20, and the Vic2a-6 bound yeast displayed the B-Victoria lineage HA0, whilst YamNGS#1 did not show any binding, which was consistent with its B-Yamagata lineage specificity (Table 3) (Figure 4). The stem binding sdAbs did not show any binding which suggested that their epitope was not accessible on the yeast cell surface.

A library of the B-Victoria lineage HA0 mutants was generated using a low rate error-prone PCR to give, approximately, 1 mutation per HA gene [16]. The library was then selected for two rounds, for the loss of binding to the B-Victoria lineage-specific sdAb Vic2a-6 (Figure 3). Random clones from the sorted population of yeast cells were sequenced to identify HA mutations that were enriched, relative to the unselected library, and so were predicted to be residues directly involved in the binding epitope. Clones with mutations introducing/replacing cysteine or proline residues were discarded, as they were predicted to have indirect effects on the sdAb binding. Several mutations were identified relative to the unselected library, which were all mapped within the 120 loop of the head domain between residues 116–137 (Figure 3), which has previously been identified as an antigenic site on the IBV-HA [29,30]. The panel of mutant HA’s was tested on the other head binding sdAbs, and mutation G133D was shown to interfere with the binding of all head-specific sdAbs, equally. Amino acid 133 sits within a highly structured region ‘PGGP’, which is consistent with having a more destabilising pleiotropic effect. The V124D mutation also interfered with all head binding sdAbs, equally, and was also classified as having a pleiotropic effect. Both of these mutations could not be associated with lineage-specific binding, so were not investigated further. The I125T mutation was also ruled out as a direct contact residue for the Vic2a-6, as it also abolished binding to all head binding sdAbs, equally. It is interesting to note that this threonine mutation was also present as a ‘naturally’ occurring variant in the early pre-lineage split strain B/HongKong/08/73, to which two of our head binding sdAbs (Vic2a-6 and Vic2a-20) did not bind (Table 2). The remaining residues 122 and 129, on the other hand, were predicted to correlate with the B-Victoria lineage binding and the enrichment of two separate mutations at position 122 (H122L and H122Y), suggested the importance of histidine 122 for the binding of Vic2a-6.

**Figure 5:**
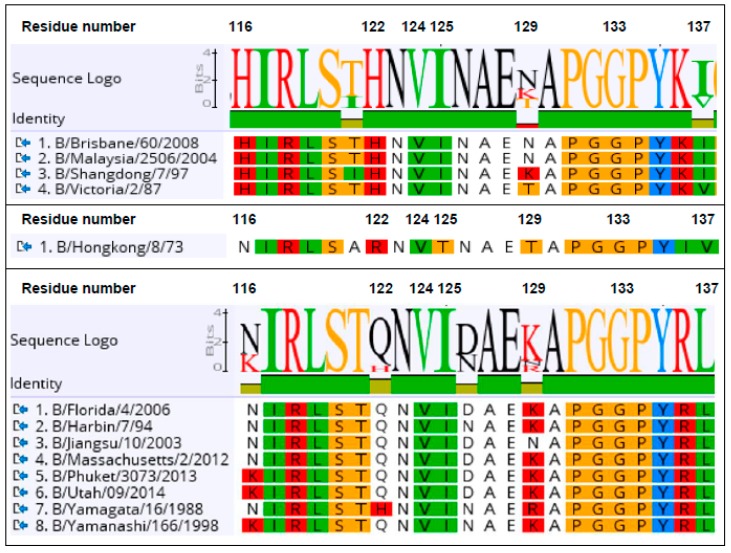
Sequence logo of antigen standards spanning predicted the epitope for lineage-specific, head-binding, single domain antibodies. Candidate residues, predicted to have a role in the binding epitope footprint of the Vic2a-6 are numbered, according to the B/HongKong/8/73 [29] within 116–137. Alignment of the B-Victoria (top) pre-lineage split strain (middle) and the B-Yamagata strains (bottom) are shown for the strains used to determine the sdAb-specificity profile (Table 2). Protein sequences used were translated from the DNA sequences: B/Brisbane/60/2008 (gb:ACN29380.1), B/Malaysia/2506/2004 (gb:ACR15732.1), B/Shangdong/9/97 (gb:AAM12546.1), B/Victoria/2/87 (gb:M58428.1) of the B-Victoria lineage; B/Yamagata/16/88 (gb:ABL77255.1), B/Yamanashi/166/98 (gb: CY019531.1), B/Harbin/7/94 (gb:AF060003.1), B/Jiangsu/10/2003 (gb:ACF54202.1), B/Florida/4/2006 (gb:ACF54246.1, B/Massachusetts/02/2012 (gb:KC892118.1), B/Phuket/3037/2013 (GISAID, EPI529345), B/Utah/9/2014 (gb|AMB72003.1) of the B-Yamagata lineage; and the pre-lineage split strain B/HongKong/8/73 (gb: AAA43717.1).

Sequence alignment of the epitope region 116–137 of the B-Victoria and the B-Yamagata strains tested in ELISA (Table 2), showed that histidine 122 and asparagine 129, spanned regions of divergence between the two lineages (Figure 5). We, therefore, re-created three single residue mutants in a wild-type HA0 background, H122L, N129D, and a naturally occurring substitution, H122Q, where the B-Vic preferred residue (histidine) was substituted with the B-Yam preferred residue (glutamine) (Figure 5). Flow cytometry experiments of the sdAb binding to the mutant HA0s (Figure 4), showed that the H122L mutation completely abolished the binding of both Vic2a-6 and Vic2a-20, whereas, the H122Q mutation reduced the binding of both sdAbs to between 14% and 24% of binding to the wt HA0, confirming the importance of this residue in the epitope footprint of both sdAbs (Figure 4). The N129D mutation only interfered with the Vic2a-6 binding, with no effect on the Vic2a-20, suggesting that, although these two sdAbs recognized the overlapping epitopes, they were not identical. This was consistent with their having unique VHH CDR3 sequences which defined their respective paratopes and interactions with HA (Table 2). None of the above three mutations had any effect on the binding of Vic2b-3, which bound to a different epitope on the HA0 head domain of the IBV.

## 4. Discussion

Monoclonal antibodies are generally seen as requiring a paired light and heavy-chain to recognise a target antigen. However, there are now several examples of stem binding human monoclonal antibodies to both IAV and IBV, which only use their heavy-chain for binding, with no contacts being made by the light chain [7,8,10]. Guided by this observation, we have previously used alpacas as a route to high-affinity, cross-neutralising, single domain antibodies (Nanobodies^TM^), naturally devoid of a paired LC, which bind to equivalent epitopes in the HA stem of the IAV [14,16]. This unique antibody format has several advantages over the conventional MAbs and, in particular, their small oblate structure and extended CDR3 loop makes them naturally equipped to access grooves and clefts, such as those on viral surfaces [21,31]. In addition, the potential for a smaller epitope footprint of the sdAbs, compared with conventional antibodies, which have evolved to bind to larger, flatter surfaces, might translate into a higher genetic barrier for an escaping virus and retention of binding for a longer period, in evolutionary time.

Within this study we have identified the sdAbs, specific to the IBV-HA, which have broad cross-reactivity and lineage-specific recognition. Single domain antibodies Vic1b-10, Yam1b-9, and the Yam2a-1 were shown to have a broad cross-reactivity, covering strains spanning over 20 years. This clearly shows that these sdAbs bind to the epitopes resistant to antigenic change and could be expected to maintain recognition of the influenza B HA, over a significant period of time (Table 2). In addition, these sdAbs were capable of neutralizing the influenza lentiviral pseudotypes, corresponding to the two prototype viruses representing the two divergent lineages, B/Victoria/2/87 and B/Yamagata/16/88, suggesting that they might have a potential in immunotherapeutics. Single domain antibodies, because of their simple molecular structure, can be easily re-formatted for half-life extension [32,33]. This could give immediate, short-term immunity, independent of influenza vaccination or the need for a functioning immune system. Longer term passive immunity, using cross-neutralising antibodies, can be achieved using a viral vector-mediated gene delivery [34,35]. In addition, the high stability and ability to withstand nebulisation are distinct advantages of the sdAbs, over human antibody formats, and mean intranasal delivery is possible, allowing deep penetration into the respiratory tract [36]. The potential applications of the cross-reactive sdAbs against IBV, extend beyond immune prophylaxis [10], and include a diagnosis of circulating IBV strains, or vaccine potency assays [19,37], for quantitating the HA content of vaccines.

Given the current requirements of the quadrivalent vaccines we sought to prove that we could isolate lineage-specific sdAbs, in a short timeframe, which would be able to maintain reactivity for a significant length of time, ideally over several seasonal vaccine changes. The identification of sdAbs, which bind to lineage-specific epitopes, is more challenging, given the high sequence and antigenic similarity between the lineages. However, using phage display and next-generation sequencing, we were able to identify, both, a B-Victoria (Vic2a-6) and a B-Yamagata (YamNGS#1) lineage-specific sdAb. Initial attempts using conventional screening to identify B-Yam lineage-specific sdAbs were unsuccessful. We reasoned that this was likely due to the low sampling of the selected clones and the dominance of the cross-reactive sdAbs during selection. Using NGS, we were able to overcome these difficulties and identify a B-Yam lineage-specific sdAb, demonstrating that NGS-assisted screening is a useful addition to the phage display process, particularly, in identifying rare sdAb specificities. The B-Victoria lineage-specific sdAb Vic2a-6 was shown to neutralise B/Brisbane/60/2008 (B-Victoria) but not the B/Florida/04/2006 (B-Yamagata), suggesting that it binds to a lineage-specific epitope in the head domain (Table 3). Another sdAb, Vic2a-20, showed preferential binding to the immobilised B-Victoria strains, using surface plasmon resonance (Table 3), with a greater than 100-fold higher affinity for the B-Victoria than the B-Yamagata HA, which was consistent with the B-Victoria lineage-specific neutralisation observed (Table 4). This suggests that the Vic2a-20 has a preferred B-Victoria lineage-specificity rather than the absolute-specificity shown by the Vic2a-6. The sdAb YamNGS#1 showed an absolute specificity for the B-Yamagata strains, with no binding to any of the B-Victoria strains tested (Table 2). The immunogen used to generate the YamNGS#1 was the 2006 B-Yamagata vaccine strain (B/Florida/04/2006) and lineage-specific binding was maintained for a period of 6 years, up to the 2012 strain (B/Massachusetts/02/2012).

**Figure 6:**
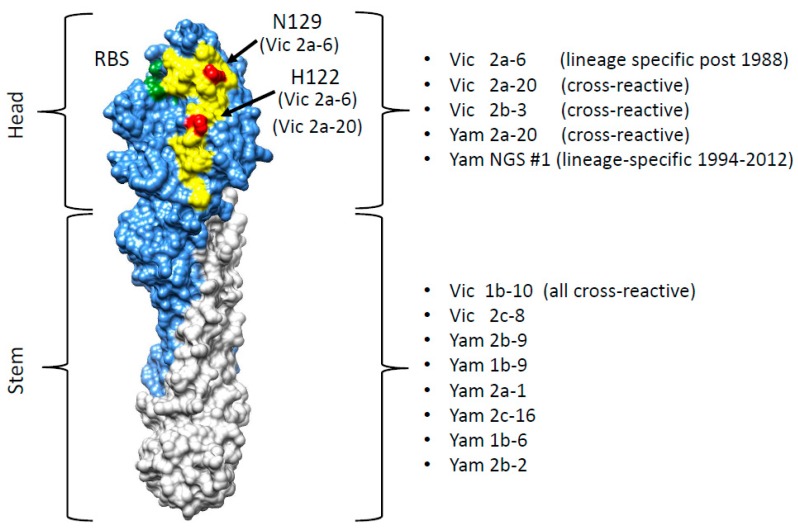
Correlation of the sdAb epitopes with the Hemagglutinin structure. The structure of the HA0 monomer (B/Brisbane/60/2008 PDB structure 4FQM) is shown with the HA1 domain in blue, the stem region in grey, the 120 loop in yellow and the receptor binding site (RBS) in green. The residue of H122 and N129 associated with the B-Victoria lineage-specific binding of the sdAb Vic2a-6 or preferred binding of the Vic2a-20 are shown in red. The binding specificity of the sdAb panel are shown in relation to the HA head and stem region.

In order to understand the structural basis of lineage-specific binding of the sdAb Vic2a-6, we used yeast display and mutational scanning [16,38]. Yeast display has emerged as a powerful tool for epitope mapping, as its eukaryotic translation machinery acts as a quality control for functional, folded, protein variants [39]. In addition, simultaneous selection for, both, display and sdAb binding, using flow cytometric cell sorting means, each protein variant can be selected on the basis of multiple parameters. Selection of a HA0 mutant library on Vic2a-6 identified several candidate mutations, predicted to specifically interfere with binding. All these mutations lie within the 120 loop spanning residues 116–137, which is a region of high antigenic diversity [29,30]. The I125T mutation was selected from our B-Victoria HA0 mutant library, which is also a naturally occurring substitution (Figure 3). Analysis of naturally occurring IBV-HA sequences identified threonine as being present in the very early pre-lineage split strain (B/Hongkong/8/73) (Figure 5), which mutated to an isoleucine in later IBV strains. The ability to relate the I125T mutation to the absence of binding of Vic2a-6 and Vic2a-20, highlighted the potential of yeast display and mutational scanning to correlate sdAb binding and strain-specificity profiles to the natural sequence divergence of IBV-HA. Mutational scanning predicted that residues 122 and 129 were key residues in the epitope footprint of Vic2a-6. The H122L mutation was shown to completely abolish binding of the Vic2a-6 and Vic2a-20, whereas N129D completely abolished binding of the sdAb Vic2a-6, with no effect on the binding of Vic2a-20. This demonstrated that although these sdAbs’ epitopes were overlapping, they were also distinct, which was consistent with the Vic2a-20 only having a ‘preferred’ B-Vic lineage-specific binding (Table 2). The naturally occurring H122Q mutation associated with the sequence divergence of IBV (Figure 5) gave a significant reduction in binding to between 14% and 24% of the wild-type HA interaction but did not completely abolish binding. This suggests that this conservative polar substitution was associated with B-Victoria lineage-specific binding, but the N129 residue was a more significant determinant of the lineage-specific binding of the Vic2a-6. The natural sequence diversity at residue 122, in the B-Victoria strains (97% His: 1% Gln), compared to the B-Yamagata strains (94% Gln: 5% His) indicated that the preferences for either amino acid, although significant, was not complete [30]. The close correlation of binding specificity in ELISA with the identity of residue 122, was highlighted by the binding of the Vic2a-6 to B/Yamagata/16/88 (Table 2) and the neutralisation of a pseudotype corresponding to this same strain (Table 4). Although this was the earliest B-Yamagata strain, following the divergence of the two lineages, it maintained a histidine at position 122, and retained binding to Vic2a-6, which was only lost in the later B-Yamagata strains with the substitution of a glutamine. Correlating the mutations which interfere with binding of the Vic2a-6 and Vic2a-20, with the structure of HA, showed that the epitope footprint was adjacent to the receptor binding site and within the 120 loop (Figure 6). The 120 loop was one of the four main regions on the influenza B HA1 head domain, identified as being dominant in the antigenic evolution of the most recent strains [29,30,40]. The identification of lineage-specific sdAbs, which are able to retain binding to an epitope, over such a long period of time (20 years in the case of Vic2a-6 and 18 years in the case of YamNGS#1), despite binding to the hyper-variable head domain, was somewhat surprising. It is interesting to speculate that this was due to the well-documented ability of sdAbs to bind to small grooves and pockets on protein surfaces, which might represent a higher genetic barrier for escape than conventional antibodies which bind to larger flatter surfaces [16].

We have shown that it is possible to isolate high-affinity, cross-reactive and lineage-specific sdAbs from alpacas immunised with a single seasonal vaccine strain, which can maintain binding and resistance to natural antigenic changes, over a significant period of time. We have also highlighted that next-generation sequencing analysis of phage displayed libraries can be useful in identifying sdAbs that may have been missed using more limited conventional ELISA-based screening. In addition, using yeast display and mutational scanning we have been able to correlate the lineage-specific binding with the structure of HA and have related natural antigenic diversity within this epitope with the sdAb (Nanobody^TM^) reagent binding. This suggests that yeast display epitope mapping could be adapted to give a comprehensive analysis of the epitope of lineage-specific sdAbs, which could be used for quadrivalent vaccine potency testing, and in addition, it could predict when a sdAb might lose its binding and needs updating [37].

## Figures and Tables

**Table 1:** VHH CDR3 sequences of specific single domain antibodies.

Nanobody	CDR3 Sequence	B-Yamagata	B-Victoria
**Vic2b-3**	AADAVVAGPDDEYDY	+	+
**Yam2a-10**	NVGFNY	+	+
**Vic2a-20**	ASKGTDYIDGIYYISYQFNS	+	+
**Vic2a-6**	VASPFSTGRATLPYQYPY	−	+
**YamNGS#1**	AAASLCSFSSNDYFY	+	−
**Yam2b-9**	AAGSGGRYDY	+	+
**Vic1b-10**	NAPTYSN	+	+
**Yam1b-9**	ALGDFTGLTNLRQAFYDF	+	+
**Yam2a-1**	NFPRSSS	+	+
**Yam2c-16**	NTHDY	+	+
**Vic2c-8**	ALGDFSGSLWAYEYDF	+	+
**Yam1b-6**	AAAKGGGAYSMISAYTY	+	+
**Yam2b-2**	RLDHWLVSGY	+	+

Binding to recombinant HA0 from the B-Victoria strain (B/Brisbane/60/2008) and the B-Yamagata strain (B/Florida/04/2006) in ELISA, indicated as binding (+) or not binding (−).

**Table 2:** Single domain antibody recognition of the whole virus antigen standards by ELISA.

Antigen Standard	Year	Vic2b-3	Yam2a-10	Vic2a20	Vic2a-6	YamNGS#1	Yam2b-9	Vic1b-10	Yam1b-9	Yam2a-1	Yam2c-16	Vic2c-8	Yam1b-6	Yam2b-2
**B/Brisbane/60/2008**	**2008**	**0.34**	**0.16**	**2.30**	**2.42**	**0.08**	**0.45**	**0.79**	**0.30**	**0.82**	**0.45**	**0.29**	**0.50**	**0.07**
**B/Malaysia/2506/2004**	**2004**	**0.07**	**0.08**	**3.54**	**1.93**	**0.09**	**0.68**	**1.39**	**0.40**	**1.43**	**0.72**	**0.40**	**0.59**	**0.07**
**B/Shangdong/7/97**	**1997**	**0.39**	**0.14**	**2.58**	**0.32**	**0.06**	**0.69**	**0.96**	**0.30**	**0.92**	**0.51**	**0.28**	**0.31**	**0.08**
**B/Victoria/2/87**	**1987**	**0.43**	**0.20**	**3.31**	**0.86**	**0.09**	**0.56**	**1.27**	**0.83**	**1.35**	**1.15**	**0.77**	**1.04**	**0.11**
**B/HongKong/8/73**	**1973**	**0.46**	**0.39**	**0.06**	**0.07**	**0.13**	**0.34**	**1.48**	**0.66**	**1.41**	**0.72**	**0.56**	**0.29**	**0.09**
**B/Yamagata/16/88**	**1988**	**0.83**	**1.76**	**2.41**	**1.07**	**0.09**	**1.59**	**1.85**	**0.98**	**1.79**	**1.01**	**0.87**	**0.71**	**0.12**
**B/Yamanashi/166/98**	**1994**	**0.74**	**1.08**	**0.11**	**0.07**	**0.07**	**1.70**	**2.16**	**1.34**	**2.41**	**1.35**	**1.24**	**1.70**	**0.14**
**B/Harbin/7/94**	**1994**	**0.28**	**0.26**	**0.14**	**0.09**	**0.44**	**0.38**	**0.57**	**0.27**	**0.52**	**0.36**	**0.27**	**0.30**	**0.06**
**B/Jiangsu/10/2003**	**2003**	**0.46**	**0.71**	**1.16**	**0.08**	**0.59**	**0.66**	**1.32**	**0.53**	**1.33**	**0.46**	**0.53**	**0.23**	**0.06**
**B/Florida/4/2006**	**2006**	**0.42**	**0.40**	**0.16**	**0.07**	**0.83**	**0.62**	**0.64**	**0.20**	**0.68**	**0.31**	**0.18**	**0.32**	**0.07**
**B/Mass’/02/2012**	**2012**	**0.53**	**0.82**	**0.79**	**0.05**	**1.00**	**3.03**	**1.82**	**0.94**	**1.96**	**0.85**	**0.84**	**1.57**	**0.10**
**B/Phuket/3073/2013**	**2013**	**0.58**	**1.22**	**0.15**	**0.06**	**0.11**	**1.01**	**0.94**	**0.55**	**0.96**	**0.45**	**0.52**	**0.32**	**0.08**
**B/Utah/9/2014**	**2014**	**0.90**	**1.36**	**0.14**	**0.07**	**0.10**	**0.31**	**1.13**	**0.52**	**1.20**	**0.43**	**0.50**	**0.21**	**0.05**
**A/Brisbane/10/2007**	**2007**	**0.05**	**0.06**	**0.07**	**0.07**	**0.07**	**0.06**	**0.06**	**0.07**	**0.06**	**0.07**	**0.06**	**0.07**	**0.07**

B-Victoria strains ranging from 1987–2008 and B-Yamagata strains from 1988–2014. Pre-lineage split strain highlighted in red. Influenza B strains used as immunogens for the sdAb library generation are highlighted in blue. Influenza A strain control highlighted in green. Table arranged to show strains moving away from pre-lineage split in time, with most recent strains being the farthest away. Values shown are the average of two independent assays. sdAb binding was tested at a single concentration of 10 µg/mL. OD_450_ ≥ 1.0, OD_450_ ≥ 0.5, OD_450_ ≥ 2 × negative control for each sdAb. B/Mass’/02/2012 is B/Massachusetts/02/2012.

**Table 3:** Epitope classification of full-length (HA0) and head domain (HA1) using surface plasmon resonance (SPR).

Single Domain Antibody	B Yam HA1 ^1^ B/Florida /4/2006	B Yam HA0 ^2^ B/Florida /4/2006	B Vic HA1 ^1^ B/Brisbane /60/2008	B Vic HA0 ^2^ B/Brisbane /60/2008	Domain	Specificity
**Vic2b-3**	0.27 nM	1.57 nM	0.06 nM	0.11 nM	Head	Cross reactive
**Yam2a-10**	3.49 nM	6.70 nM	+ ^3^	3.30 nM	Head	Cross reactive
**Vic2a-20**	173 nM	-	0.79 nM	0.48 nM	Head	Cross reactive
**Vic2a-6**	-	-	0.07 nM	0.08 nM	Head	B-Vic lineage
**YamNGS#1**	11.8 nM	2.39 nM	-	-	Head	B-Yam lineage
**Yam2b-9**	-	2.13 nM	-	10.0 nM	Stem	Cross reactive
**Vic1b-10**	-	0.28 nM	-	1.1 nM	Stem	Cross reactive
**Yam1b-9**	-	0.10 nM	-	0.08 nM	Stem	Cross reactive
**Yam2a-1**	-	0.14 nM	-	7.40 nM	Stem	Cross reactive
**Yam2c-16**	-	1.46 nM	-	0.76 nM	Stem	Cross reactive
**Vic2c-8**	-	0.13 nM	-	0.37 nM	Stem	Cross reactive
**Yam1b-6**	-	5.43 nM	-	0.60 nM	Stem	Cross reactive
**Yam2b-2**	-	10.0 nM	-	1.36 nM	Stem	Cross reactive

^1^ HA1 is purified hemagglutinin head domain (Residues Met1-Arg361 of B/Florida/04/2006 and residues Met1-Arg362 of B/Brisbane/60/2008). ^2^ HA0 is purified full-length hemagglutinin. ^3^ Binding could be seen but could not be resolved using the BIAevaluation^TM^ software.

**Table 4:** Neutralisation of the influenza B pseudotype viruses.

Single Domain Antibody	B/Yamagata /16/1988 (Yamagata) IC_50_ [nM]	B/Victoria /2/1987 (Victoria) IC_50_ [nM]	B/Florida /4/2006 (Yamagata) IC_50_ [nM]	B/Brisbane /60/2008 (Victoria) IC_50_ [nM]
**Vic2b-3**	104	81	246	240
**Yam2a-10**	-	-	-	-
**Vic2a-20**	-	1.1	-	112
**Vic2a-6**	315	159	-	10
**YamNGS1#1**	-	-	NT	NT
**Yam2b-9**	30.5	-	NT	NT
**Vic1b-10**	0.2	15.5	NT	NT
**Yam1b-9**	31.7	81.8	NT	NT
**Yam2a-1**	81.8	6	NT	NT
**Yam2c-16**	NT	NT	NT	NT
**Vic2c-8**	202.5	228	NT	NT
**Yam1b-6**	26.6	12.6	NT	NT
**Yam2b-2**	-	113.5	NT	NT

NT—Not Tested; IC_50_ is the sdAb concentration in nM that gives 50% inhibition; and (-) indicates no neutralisation activity.

## Supplementary Materials

The following are available online at https://www.mdpi.com/2073-4468/8/1/14/s1, Figure S1: Isolation of the B-Yamagata lineage-specific nanobodies, using NGS. High copy number CDR3’s using antibody mining toolbox (A were normalised for each sequencing run and presented as % Relative Frequency (B,C). Fold increase in CDR3 frequencies were then calculated from a CDR3 frequency, in the unselected phage library and the same CDR3’s frequency after selection of the HA1 domain, or of the full-length HA0 (D,E). Figure S2: Grouping as head- or stem-specific binding using surface plasmon resonance (SPR). The kinetic binding constants (*k**on* and *k**off*) of the panel of nanobodies were determined using SPR and single-cycle kinetics. Data are presented as rate plots with iso-affinity diagonals (RAPID) where the diagonals (dotted lines) are connecting the points of equal dissociation constant (KD). Affinity on (A) the B-Yamagata head domain (HA1) of the B/Florida/04/2006, (B) the full-length HA0 of the B/Florida/04/2006, (C) the B-Victoria head domain (HA1) of the B/Brisbane/60/2008, and (D) the full length HA0 of the B/Brisbane/60/2008, is shown. Fitting was with single-cycle kinetics and a 1:1 Langmuir fitting model, using the BIAevaluation^TM^ software (GE Healthcare, Marlborough, MA, USA). Equilibrium dissociation constants are given in Table 3.
