# Supplementary material for: Cross-Reactive and Lineage-Specific Single Domain Antibodies against Influenza B Hemagglutinin
Source: Antibodies (Basel). 2019 Feb 10;8(1):14. doi: 10.3390/antib8010014 (PMC6640691; doi:10.3390/antib8010014)
Supplement: Supplementary file 1 [file antibodies-08-00014-s001.pdf]

A

	CDR3 copy number			
Candidate CDR3's	B-Yamagata Phage library	Round 1 B-Yamagata HA1	Round 1 B-Yamagata HA0	Round 1 B-Victoria HA0
**NGS#1 AAASLCSFSSNDYFY**	15	1666	0	135
**NGS#2 ATGCPRNGSYYHTGY**	243	3078	276	70
**NGS#3 ADDCSGPVWGS**	10	2213	68	19
**NGS#4 TTDWSTYCDLGPRKYNK**	1	244	6	3
**Total CDR3s**	**26504**	**30270**	**35037**	**40633**

B

	CDR3 % relative frequency			
Candidate CDR3's	B-Yamagata Phage library	Round 1 B-Yamagata HA1	Round 1 B-Yamagata HA0	Round 1 B-Victoria HA0
**NGS#1 AAASLCSFSSNDYFY**	0.057	5.504	0.000	0.332
**NGS#2 ATGCPRNGSYYHTGY**	0.917	10.168	0.788	0.172
**NGS#3 ADDCSGPVWGS**	0.038	7.311	0.194	0.047
**NGS#4 TTDWSTYCDLGPRKYNK**	0.004	0.806	0.017	0.007

C

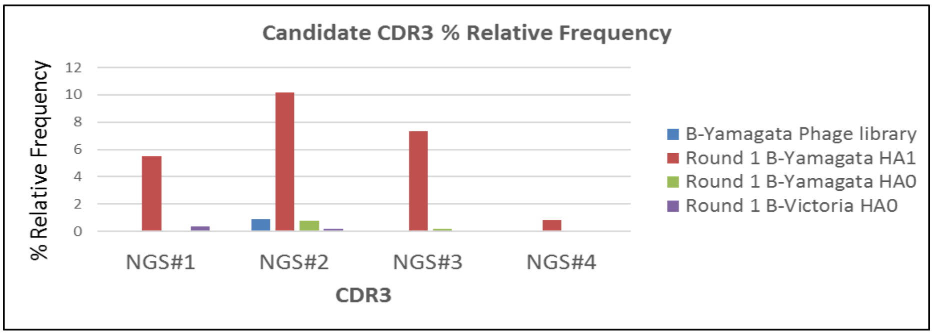

D

	CDR3 fold enrichment			
Candidate CDR3's	B-Yamagata Phage library	Round 1 B-Yamagata HA1	Round 1 B-Yamagata HA0	Round 1 B-Victoria HA0
**NGS#1 AAASLCSFSSNDYFY**	NA	97.248	0.000	5.870
**NGS#2 ATGCPRNGSYYHTGY**	NA	11.091	0.859	0.188
**NGS#3 ADDCSGPVWGS**	NA	193.767	5.144	1.239
**NGS#4 TTDWSTYCDLGPRKYNK**	NA	213.643	5.000	1.957

E

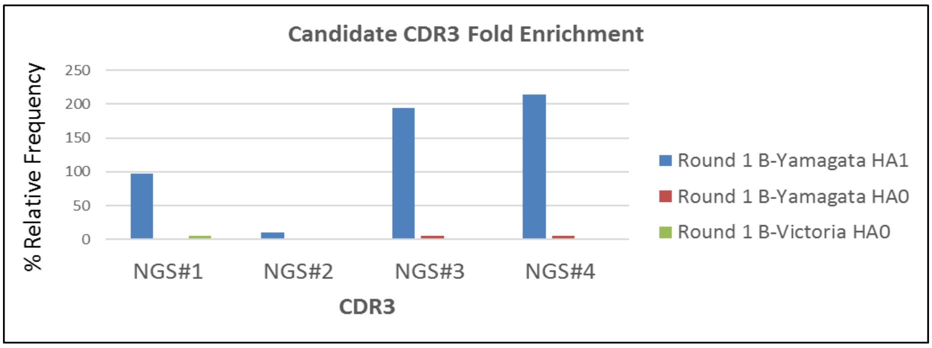

Supplementary Figure S1. Isolation of B-Yamagata lineage specific nanobodies using NGS. High copy number CDR3's using antibody mining toolbox (A) were normalised for each sequencing run and presented as % Relative Frequency (B, C). Fold increase in CDR3 frequencies were then calculated from a CDR3 frequency in the unselected phage library and the same CDR3's frequency after selection on the HA1 domain or on full length HA0 (D, E).

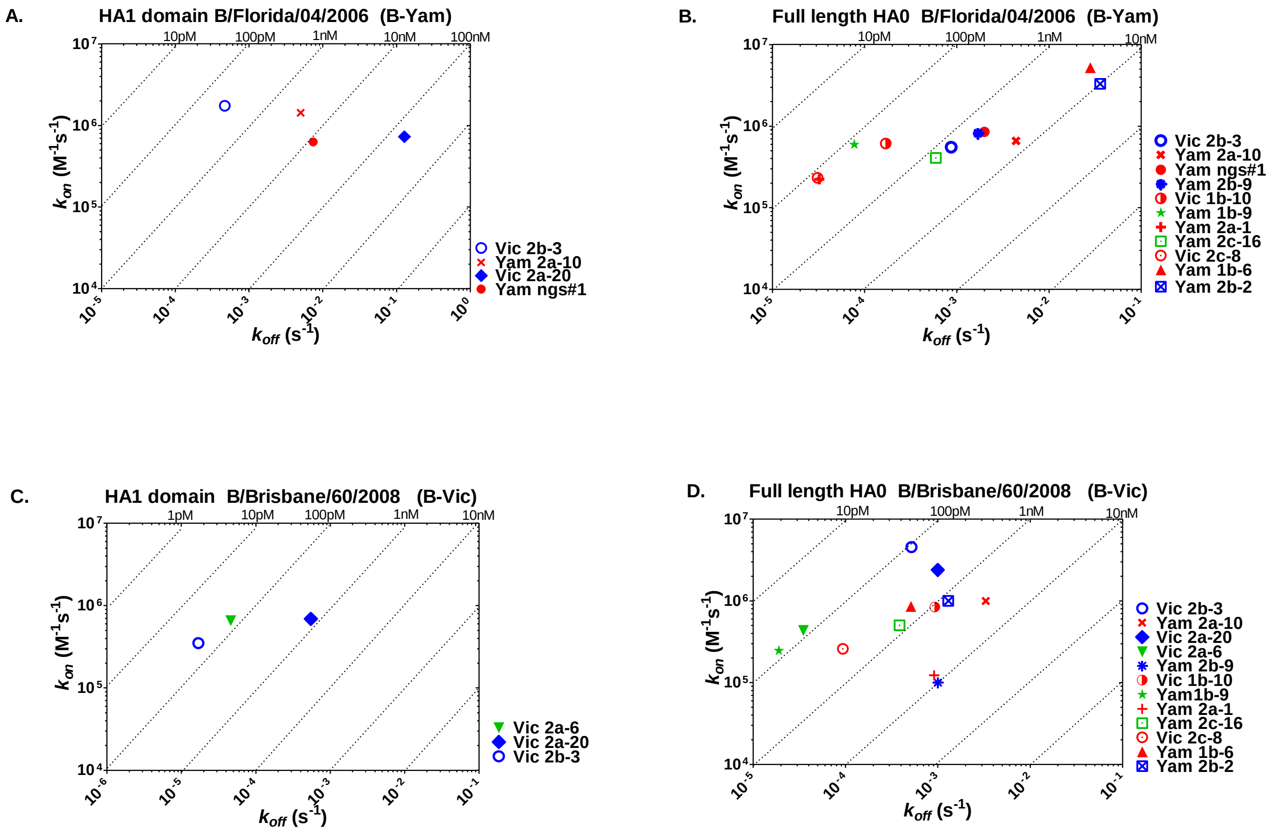

Supplementary Figure S2. Grouping as head or stem specific binding using SPR. The kinetic binding constants (*k_{on} and k_{off}*) of the panel of nanobodies were determined using SPR and single cycle kinetics [22]. Data are presented as rate plots with iso-affinity diagonals (RAPID) where the diagonals (dotted lines) are connecting the points of equal dissociation constant (K_D). Affinity on (A) B-Yamagata head domain (HA1) of B/Florida/04/2006, (B) full length HA0 of B/Florida/04/2006, (C) B-Victoria head domain (HA1) of B/Brisbane/60/2008 and (D) full length HA0 of B/Brisbane/60/2008 is shown. Fitting was with single cycle kinetics and a 1:1 Langmuir fitting model using BIAevaluation™ software. Equilibrium dissociation constants are given in Table 3.
